# Unveiling the Mineral and Sugar Richness of Moroccan Honeys: A Study of Botanical Origins and Quality Indicators

**DOI:** 10.3390/molecules30010150

**Published:** 2025-01-02

**Authors:** Azzedine Abeslami, Hammadi El Farissi, Francesco Cacciola, Ali El Bachiri, Mariane Sindic, Marie-Laure Fauconnier, Etienne Bruneau, Abdelmonaem Talhaoui

**Affiliations:** 1 Laboratory of Environment and Applied Chemistry (LCAE), Team: Physical Chemistry of the Natural Resources and Processes, Faculty of Sciences, Mohammed First University, Oujda 60000, Morocco; abeslami.azzedine@gmail.com (A.A.); a.elbachiri@ump.ac.ma (A.E.B.); a.talhaoui@ump.ac.ma (A.T.); 2 Chemical Engineering for Resources Valorization Group (UAE/L01FST), Faculty of Sciences and Technology, Abdelmalek Essaadi University, Tangier 90010, Morocco; 3 Messina Institute of Technology c/o, Department of Chemical, Biological, Pharmaceutical and Environmental Sciences, Former Veterinary School, University of Messina, Viale G. Palatucci snc, 98168 Messina, Italy; 4 Laboratory of Agro-Food Quality and Safety, Faculty of the Agronomic Sciences, Gembloux Agro-Bio Tech, University of Liège, Beekeeping Research and Information Centre (CARI), 1348 Louvain-la-Neuve, Belgium; marianne.sindic@ulg.ac.be; 5 Laboratory of Chemistry of Natural Molecules, Gembloux Agro-Bio Tech, Liege University, Passage des Déportés 2, 5030 Gembloux, Belgium; marie-laure.fauconnier@uliege.be; 6 Beekeeping Research and Information Centre (CARI), 1348 Louvain-la-Neuve, Belgium; bruneau@cari.be

**Keywords:** honey, mineral content, heavy metals, botanical origin, principal component analysis, sugar profile

## Abstract

This study comprehensively analyzes the mineral and heavy metal profiles of seven honey types, focusing on the contents of potassium (K), calcium (Ca), magnesium (Mg), iron (Fe), zinc (Zn), manganese (Mn), copper (Cu), cadmium (Cd), and lead (Pb), with particular emphasis on honey produced in eastern Morocco. Multifloral honey was found to have the highest total mineral content (661 mg/kg), while rosemary honey had the lowest (201.31 mg/kg), revealing the strong influence of floral and botanical origin. Darker honey, such as multifloral and jujube, were richer in minerals, with potassium consistently being the most abundant, followed by calcium, magnesium, and iron, while cadmium and lead remained within safe, trace-level concentrations. Additionally, sugar profiling showed that all samples contained fructose, glucose, maltose, turanose, erlose, sucrose, and palatinose, with particularly high fructose and glucose contents in multifloral honey. Principal component analysis (PCA) accounted for 75% of the variation and identified three distinct groups of honey based on mineral content multifloral, eucalyptus, and rosemary. Multifloral and eucalyptus honey had higher concentrations of iron, magnesium, and calcium, whereas rosemary honey was richer in zinc and copper. The findings underscore the potential of honey as a marker of environmental quality and suggest that eastern Morocco honey possesses favorable characteristics for national and international commercialization.

## 1. Introduction

Eastern Morocco holds substantial potential for producing high-quality honey varieties due to its rich diversity of medicinal and aromatic plants, such as thyme, rosemary, carob, and jujube. These plants contribute to honey’s unique chemical composition, endowing it with various bioactive metabolites that attract consumers seeking natural, health-promoting foods. While sugars dominate honey’s composition, it also contains numerous bioactive compounds, including natural antioxidants, which contribute to its biological activity and enhance its nutritional profile [1]. The mineral content, determined by the plant source, is a key factor in distinguishing honey types and helps to indicate the origin and botanical sources of honey [2]. Typically, the mineral content in honey ranges from 0.04% to 0.2%, with darker honey varieties generally having higher mineral levels than lighter ones [3,4]. 

The main elements found in honey can be classified into two categories: major (macro) elements such as Na, K, Ca, Mg, P, S, and Cl, and trace elements, including a variety of heavy metals like Al, Cu, Pb, Zn, Mn, Cd, Tl, Co, Ni, Rb, Ba, Be, Bi, U, V, Fe, Pt, Pd, Te, Hf, Mo, Sn, Sb, La, I, Sm, Tb, Dy, Sd, Pr, Nd, Tm, Yb, Lu, Gd, Ho, Er, Ce, Cr, As, B, Br, Hg, Se, and Sr. No single honey sample has been shown to contain all these elements, and most studies have identified specific mineral groups depending on a honey’s floral and geographical origin [5,6,7]. However, the presence of heavy metals in honey poses serious health risks, such as multiple sclerosis, Alzheimer’s, and Parkinson’s disease [8], and may even contribute to psychological and neurological disorders, including autism [9]. Thus, evaluating the mineral content of honey is crucial, not only to assess its nutritional and medicinal properties but also to ensure its safety for human consumption [10].

Despite variations in honey composition based on the plants bees visit, the primary constituents, such as fructose and glucose, remain consistent across honey types [11]. These sugars are central to honey’s characteristics, particularly influencing its crystallization behavior [12]. Sucrose, while present in smaller amounts, can serve as an indicator of honey ripeness. High sucrose content typically suggests an early harvest, as the sucrose has not fully converted to fructose and glucose due to incomplete invertase enzyme activity [13]. Reliable honey samples generally contain less than 5% sucrose [14]. Over time, honey tends to crystallize, with glucose precipitating as glucose monohydrate, resulting in a more stable, saturated solution [15,16]. The fructose/glucose (F/G) ratio is a critical factor in determining crystallization tendencies, with a low F/G ratio typically indicating greater resistance to crystallization [17,18]. In addition to its beneficial constituents, honey can also be contaminated with various chemicals, such as pesticides, antibiotics, and heavy metals, which compromise its health benefits [19,20]. Studies on Moroccan honey from regions like Fez-Meknes and Middle Atlas, have revealed valuable insights into its physicochemical, antioxidant, and antibacterial characteristics [21,22]. However, due to the potential for contamination, producing honey free of harmful substances is essential to maintain its nutritional and therapeutic integrity [23].

Despite the known benefits of honey and its importance as a dietary component, there is limited information on the chemical composition of honey produced in eastern Morocco [24]. While honey from other Moroccan regions has been studied, the specific sugar and mineral profiles of eastern Moroccan honey, covering varieties such as jujube, multifloral, citrus, eucalyptus, thyme, carob, and rosemary, remain unexplored. Understanding these profiles is essential for establishing quality standards and assessing potential health risks due to toxic contaminants.

This study addresses the gap in knowledge regarding the composition of eastern Moroccan honey. By analyzing the sugar content and mineral composition of several types of honey from this region, this research aims to provide a comprehensive understanding of their nutritional and health-related properties. Furthermore, the findings will allow for comparisons with honey from other regions worldwide, contribute to setting regional quality standards, and evaluate potential toxicological risks. This work will thus support the promotion of eastern Moroccan honey as a nutritious, high-quality food product that can meet safety and health standards. 

## 2. Results and Discussion

### 2.1. Sugar Content

The sugar composition analysis of the studied honey samples is detailed in Table 1. A total of ten sugars were identified and quantified using GC-FID (Table S1, Figures S1–S7), comprising two monosaccharides, three disaccharides, and five trisaccharides. Significant statistical differences were observed in the sugar compositions among the various honey groups evaluated (*p* < 0.05). Multifloral honey exhibited the highest total sugar content at 83.67%, while eucalyptus honey had the lowest at 68.02%. The total sugar content in eastern Moroccan honey (83.67%) was higher than that reported for honey from Algeria [25]. The sugar profile in honey is primarily influenced by its botanical and geographical origins, alongside factors such as weather, processing, and storage conditions [26,27,28]. Among the analyzed samples, glucose and fructose were the predominant carbohydrates, with fructose being the major sugar, followed by glucose, maltose, turanose, erlose, sucrose, and palatinose. Fructose concentrations ranged from 33.88% to 40.14%, while glucose levels varied between 26.52% and 35.69%, with an average glucose concentration of 29.97%. The total monosaccharide content of glucose and fructose (F + G) fell within the European Union’s authorized limits (>60%), with the highest cumulative value recorded in *multifloral* honey (75.83%). This value surpassed findings by Kuan Wei Se [29] and Siok Peng Kek [30], who reported (F + G) values of 29.8% and 24.99%, respectively. In contrast, Chuttong et al reported lower (F + G) contents (31.0%) in honey samples from Thailand [31]. Reducing sugars such as maltose palatinose, and turanose were also analyzed. Maltose content ranged from 3.16% (*Carob* honey) to 5% (*Citrus* honey), with significant differences (*p* < 0.05) between citrus honey and other types, except for multifloral honey (*p* > 0.05). The turanose content varied from 0.85% (*Eucalyptus* honey) to 1.80% (*Jujube* honey), again showing significant differences (*p* < 0.05) compared to other honey types. Palatinose content ranged from 0.05% (carob honey) to 0.68% (jujube honey), with significant differences observed between jujube honey and other varieties. Maltose, turanose, and palatinose were present in all the honey samples, with maltose levels consistent with findings by Ouchmoukh et al. [25], while the turanose and palatinose results were similar to those reported by Guenaoui et al. [32] and De la Fuente et al. [33]. For sucrose concentration, the Codex Alimentarius Commission (2001) establisheds a limit of 5% to ensure the purity and quality of honey samples. Excess sucrose can elevate blood sugar levels, and the current study found sucrose concentrations in eastern Moroccan honey ranging from 0.15% to 1%. The non-reducing trisaccharides, erlose, raffinose, and panose, were also measured, with erlose present in all honey samples and ranging from 0.22% to 1.64%, which is higher than previously reported by Pérez-Arquillué et al. [34]. Eucalyptus honey exhibited the highest erlose concentration.

Panose was detected in jujube and multifloral honey at very low levels, with mean values not exceeding 0.05%. Another crucial factor influencing honey quality is the ratio of fructose to glucose (F/G), which indicates its crystallization potential. A higher F/G ratio suggests that honey will remain liquid, as glucose is less soluble in water compared to fructose, making it more prone to crystallization [35]. Honey crystallization slows when the F/G ratio exceeds 1.3 and accelerates when it drops below 1.0. In this study, the F/G ratio ranged from 1.0 to 1.35. Jujube, citrus, and thyme honeys had lower glucose amounts (28.09%, 29.17%, and 26.52%, respectively) and higher fructose proportions (36.92%, 38.51%, and 35.95%, respectively), keeping these honey types in a liquid state due to their F/G ratios exceeding 1.30% [25].

Beyond affecting sensory characteristics and physical states, the F/G ratio is an important criterion for honey’s application in managing physiological conditions like lipid and glucose metabolic dysfunctions. Research by Pasupuleti et al. [36] indicated that fructose in honey could improve hyperglycemia in diabetic animals and patients. Moreover, dietary fructose has been shown to enhance glycemic status by increasing glucokinase activity, facilitating the conversion of glucose to glucose-6-phosphate, and promoting hepatic glucose uptake [37]. Thus, fructose-rich honey may offer benefits in enhancing human physiological function and preventing metabolic disorders such as diabetes.

### 2.2. Mineral Content

Nine metals were quantified in each honey sample: potassium (K), calcium (Ca), magnesium (Mg), iron (Fe), zinc (Zn), manganese (Mn), copper (Cu), cadmium (Cd), and lead (Pb). The mean concentrations of these minerals across seven honey types, expressed in mg/kg of fresh weight, are detailed in Table 2. The mean total mineral content ranged significantly from 661 mg/kg in multifloral honey to 201.31 mg/kg in rosemary honey. In comparison to multifloral and jujube honey, the rosemary, carob, and thyme honeys exhibited lower mineral levels, indicating that the mineral content in honey is strongly influenced by the floral sources utilized by the bees. Various factors, such as geographical location, climate, seasons, flower types, and soil composition, affect the mineral content in honey [38]. Darker honey varieties are typically richer in minerals, a characteristic also shared by multifloral and jujube honey samples in the study by Chua [39]. The results for the nine elements varied significantly based on the botanical origin of the honey, revealing three distinct groups of mineral concentrations. The first group consists of primary metals, with potassium being the most abundant, comprising 67.65% of the total mineral content; this finding aligns with other studies that highlight potassium as the predominant element in honey [1]. Calcium, the second most abundant element, accounted for 20.52%, followed by magnesium at 6.89% and iron at 2.66%. The second group comprises minor elements present in all honey types, including copper, manganese, and zinc, with average concentrations of 1.52 mg/kg, 2.53 mg/kg, and 4.57 mg/kg, respectively. The third group includes trace elements such, as cadmium and lead, with average concentrations ranging from 0.00075 to 0.14 mg/kg. Overall, potassium, calcium, and magnesium were the most abundant minerals in the honey samples analyzed, which is consistent with findings regarding honey from the Canary Islands, Spain [40].

In examining potassium content across different honey samples, the highest concentration (452.66 mg/kg) was found in multifloral honey, while the lowest (49.31 mg/kg) was found in rosemary honey, with a statistically significant difference (*p* < 0.05) between these values. This trend is consistent with other studies that report potassium as the most abundant mineral in honey from various regions, including Spain, Poland, Slovenia, Portugal, and Italy [41,42,43,44]. Calcium levels varied between 12.33 mg/kg and 145.24 mg/kg, surpassing findings by Rizelio [45] and Czipa [46] who reported lower ranges for *Apis mellifera* honey. Furthermore, jujube honey was identified as the best source of magnesium and zinc, with average contents of 38.66 mg/kg and 5.50 mg/kg, respectively.

Regarding heavy metals, lead levels in the analyzed honey samples ranged from 0.09 mg/kg in thyme honey to 0.19 mg/kg in multifloral honey. These values were lower than the average lead concentration (0.22 mg/kg) reported for various Algerian honeys by Dalila Bereksi-Reguig [47] and exceeded the Codex Alimentarius and European Union standards, which set a limit at 0.3 mg/kg. Cadmium concentrations ranged from 0.0014 mg/kg to 0.018 mg/kg, remaining below the European limit of 0.05 mg/kg (Codex Alimentarius, 2000, [48]). Despite the detection of heavy metals, their concentrations in all analyzed samples were within safe limits for consumption. Honey can be contaminated through various sources, including environmental pollutants, such as heavy metals and pesticides, often due to non-hygienic practices during harvesting and processing [49]. As such, honey serves as a useful indicator of environmental pollution [50], with all analyzed samples considered safe for consumption based on the heavy metal levels detected.

### 2.3. Principal Component Analysis (PCA)

**Figure 1:**
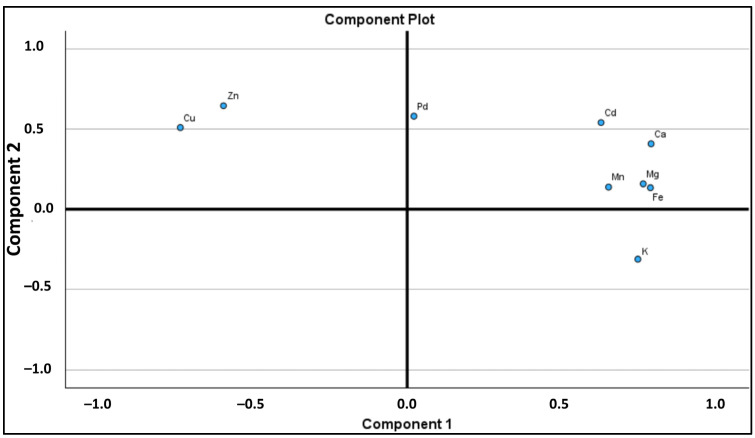
Loading plots of the mineral contents of honey samples at principal components PC1 and PC2.

The principal component analysis (PCA) (Table 3) results provide a comprehensive understanding of the relationships among elements in honey samples, encapsulated in two main components. PC1 accounts for 56.143% of the total variance, with an eigenvalue of 5.031, and is characterized by strong positive loadings for calcium (Ca: 0.794), iron (Fe: 0.791), magnesium (Mg: 0.768), manganese (Mn: 0.655), and potassium (K: 0.751). This suggests that these elements tend to vary together, likely due to similar environmental influences. Conversely, copper (Cu: −0.738) and zinc (Zn: −0.598) exhibit strong negative loadings, indicating an inverse relationship with the positively loaded elements, possibly resulting from competitive environmental or chemical factors. PC2, with an eigenvalue of 1.751, explains an additional 19.586% of the variance, bringing the cumulative explained variance to 75.730%. It highlights distinct associations among elements such as zinc (Zn: 0.645), palladium (Pd: 0.579), cadmium (Cd: 0.540), and copper (Cu: 0.509), suggesting these may stem from different contamination sources. The clustering of multifloral and eucalyptus honeys in the upper right of the score plot (Figure 1) indicates high concentrations of Fe, Mg, and Ca, while rosemary honey, positioned with positive PC1 and negative PC2 scores, reflects elevated levels of Zn and Cu alongside lower potassium (K: −0.311) concentrations. This analysis underscores the complexity of mineral interactions and their implications for the quality and authenticity of honey, as well as the potential health benefits associated with different types.

**Figure 2:**
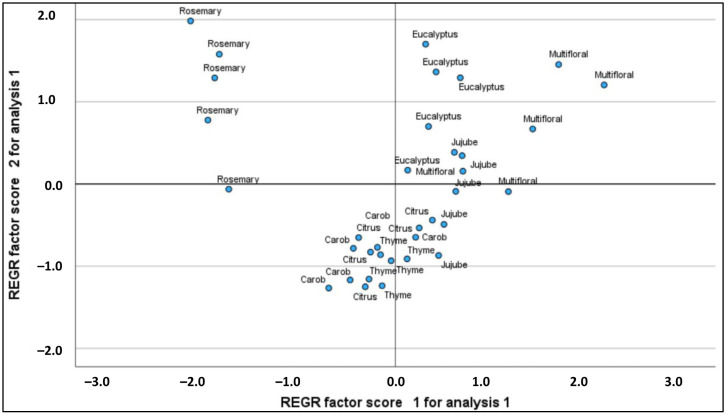
Score plot of the 35 honey samples.

The score plot presented in Figure 2 for the 35 honey samples illustrates distinct differences among the various types of honey, which are likely attributed to their analytical characteristics derived from a principal component or factor analysis. The x-axis (REGR factor score 1) and y-axis (REGR factor score 2) represent two primary factors summarizing the variability of the samples, with values ranging from −3 to +3 for the x-axis and −2 to +2 for the y-axis. Each point corresponds to a sample identified by its type (e.g., “Rosemary”, “Eucalyptus”, “Multifloral”, etc.), and their position indicates a similarity or difference according to these factors.

Rosemary honeys are grouped in the upper left quadrant, with x-scores close to −2 and y-scores between 1 and 2, indicating distinct and homogeneous characteristics. Eucalyptus honeys are predominantly located in the right half of the graph, with positive x-values (between 1 and 3) and y-values (around 1), illustrating a homogeneous profile that differs from that of rosemary. Multifloral honeys appear on the right side, with x-values ranging from 0.5 to 3 and y-values around 0, showing wider dispersion that suggests variability due to floral diversity.

In the bottom left and center, jujube, carob, citrus, and thyme honeys are found with x-values ranging from −1 to 1 and y-values from −1 to 0.5, indicating similarities with less internal variability compared to multifloral honeys. The distance between the points represents the similarity of the samples: points close to each other, such as those for honeys of the same type, indicate similar compositions, while those further apart demonstrate marked differences. This graph highlights the unique chemical profiles and proximities of honeys according to their botanical origins, suggesting the analysis’s usefulness for authentication or classification based on floral origin.

## 3. Materials and Methods

### 3.1. Collection and Preparation of Honey Samples

Thirty-five honey samples, including jujube, multifloral, citrus, eucalyptus, thyme, carob, and rosemary, were obtained from beekeepers from eastern Morocco, specifically from Oujda (Angad), Berkane (Tafoughalt), Jerada (Guenfouda), Nador (Aroui), and Taourirt (Naïma) (Table S2). The types of honey collected Each sample was stored in a sealed plastic container at room temperature (22–24 °C) to maintain its original composition until further analysis.

### 3.2. GC-FID Determination of Sugars

The sugar content of the honey samples was determined using gas chromatography with flame ionization detection (GC-FID), following the Pierce Portallier method by Bagdanov [51]. First, 3 g of each honey sample was diluted in demineralized water and heated to 50 °C to expedite dissolution. The solution was then transferred to a 500 mL volumetric flask, topped with demineralized water, and stirred for 10 min to ensure homogeneity. A 100 µL aliquot of each honey solution was combined with 100 µL of mannitol in Eppendorf tubes, then air-dried and dehydrated. Following this, 200 µL of an oximation solution was added, with the mixtures homogenized and heated at 65 °C while stirring at 1400 rpm for 15 min to form oximes. These oximes were then silylated with 100 µL of hexamethyldisilazane and 10 µL of trifluoroacetic acid at 25 °C for 30 min. After preparation, 200 µL of each solution was injected into a Hewlett Packard 6890 series gas chromatograph equipped with an FID detector and an HP1-methylsiloxane capillary column (30 m × 320 µm, 0.25 µm). The GC conditions were as follows: the initial oven temperature was set to 70 °C, followed by a temperature increase at a rate of 49 °C/min from 70 °C to 140 °C and then at a rate of 6 °C/min from 140 °C to 300 °C. Helium (99.99%) was used as the carrier gas, with a flow velocity of 1 mL/min. Each sample was analyzed twice for precision, and the average peak areas were used for quantification. Standards for sugar analysis included glucose, fructose, sucrose, maltose, turanose, melibiose, palatinose, trehalose, palatinose, raffinose, erlose, melezitose, maltotriose, and panose.

### 3.3. Analysis of the Mineral Content

To analyze mineral content, 0.5 g of each honey sample was transferred to a 50 mL conical flask, and 5 mL of aqua regia (3.5 mL of hydrochloric acid and 1.5 mL of nitric acid) was added. Aqua regia was chosen for its strong oxidative properties, ensuring the complete digestion of the organic matrix and the removal of any interfering compounds, which is critical for ac-curate AAS analysis. The mixture was heated under reflux for 2 h to facilitate the breakdown of organic matter and the release of mineral ions into the solution. After cooling, the digested sample was filtered to remove any residual solids, ensuring a matrix-free solution. The filtrate was then diluted to a final volume of 50 mL with distilled water, making it ready for analysis by an atomic absorption spectrometer (Perkin Elmer AAS AAnalyst 800, Shelton, CT, USA). This preparation method aligns with standard AAS protocols, where complete matrix removal is essential for minimizing spectral and chemical interferences. Standardized analytical parameters included the radio frequency power (0.7–1.5 kW, with 1.2–1.3 kW for axial measurements), plasma gas flow rate (argon; at 10.5–15 L/min for radial and 15 L/min for axial measurements), auxiliary gas flow rate (argon; at 1.5 L/min), and a viewing height of 5–12 mm [52].

### 3.4. Statistical Analysis

The results were expressed as a mean ± standard deviation derived from triplicate analyses for all measurements. To evaluate variations in the observed parameters among the honey samples, an analysis of variance (ANOVA) was conducted to compare the means. Furthermore, unsupervised principal component analysis (PCA) was utilized with IBM SPSS Statistics 30.0.0 to classify the honey samples based on their mineral content.

## 4. Conclusions

This study examined the sugar and mineral content of seven honey varieties from eastern Morocco, providing valuable insights into their nutritional profiles and botanical origins. Sugar analysis revealed that all samples predominantly contained reducing sugars, particularly fructose and glucose, with *multifloral* honey exhibiting the highest levels of these sugars, as well as sucrose, while jujube honey had the highest concentrations of turanose and palatinose. The high fructose-to-glucose ratio (ranging from 1 to 1.35) contributed to slower crystallization, providing a unique texture characteristic of certain honey types. In terms of mineral composition, nine elements were analyzed, with potassium being the most abundant, comprising 67.65% of the total mineral content. *Multifloral* honey had the highest mineral levels (661 mg/kg), whereas viper *rosemary* honey had the lowest (201.31 mg/kg). The low concentrations of toxic metals, like cadmium and lead, suggest that the honeys were produced in a clean environment. Their richness in essential minerals, such as calcium, iron, and magnesium, indicates a high nutritional value, supporting the health benefits associated with these honeys.

Principal component analysis (PCA) revealed that mineral composition effectively differentiates honey types based on their botanical origins. The PCA results demonstrated that elements like calcium, iron, magnesium, manganese, and potassium tended to cluster together, while zinc and copper were inversely related to these elements. This clustering pattern suggests that the mineral content of honey is influenced by similar environmental factors, contributing to the unique profiles of each honey type. The clustering of honey types in the score plot further highlighted these differences, with multifloral and eucalyptus honeys exhibiting higher concentrations of essential minerals, while rosemary honey had elevated levels of zinc and copper. These findings emphasize the importance of mineral composition in the classification and authenticity of honey, as well as its potential health benefits.

Overall, the study affirms the exceptional quality of Moroccan honeys, enhancing their appeal in national and international markets. The results provide valuable insights into their environmental implications and nutritional value. Future research could investigate how various environmental factors, such as climate and soil composition, influence the mineral and sugar profiles of honey, thereby deepening our understanding of the elements that shape the distinct characteristics of these products.

## Figures and Tables

**Table 1:** Sugars values (%) of honey types.

Honey Type	Jujube	Multifloral	Citrus	Eucalyptus	Thyme	Carob	Rosemary
Sugar	Composition						
**Fructose (%)**	36.92 ± 0.73 ^b^	40.14 ± 0.32 ^a^	38.51 ± 0.37 ^a^	34.44 ± 0.25 ^a^	35.95 ± 0.45 ^b^	38.56 ± 0.76 ^b^	33.88 ± 0.38 ^a^
**Glucose (%)**	28.09 ± 0.31 ^a^	35.69 ± 0.39 ^a^	29.17 ± 0.45 ^b^	27.02 ± 0.28 ^a^	26.52 ± 0.45 ^b^	29.62 ± 0.49 ^b^	33.82 ± 0.54 ^b^
**Maltose (%)**	3.96 ± 0.09 ^c^	4.23 ± 0.16 ^a^	5 ± 0.22 ^a^	3.66 ± 0.12 ^a^	4.88 ± 0.38 ^a^	3.16 ± 0.23 ^a^	3.52 ± 0.39 ^a^
**Turanose (%)**	1.80 ± 0.29 ^a^	1.49 ± 0.12 ^a^	1 ± 0.36 ^a^	0.85 ± 0.28 ^a^	1.48 ± 0.34 ^a^	0.93 ± 0.27 ^a^	0.99 ± 0.13 ^a^
**Erlose (%)**	1.58 ± 0.06 ^c^	0.63 ± 0.01 ^c^	0.22 ± 0.03 ^c^	1.64 ± 0.05 ^c^	0.52 ± 0.02 ^c^	0.37 ± 0.04 ^c^	0.59 ± 0.02 ^c^
**Palatinose (%)**	0.68 ± 0.09 ^c^	0.38 ± 0.06 ^c^	0.32 ± 0.04 ^c^	0.26 ± 0.05 ^c^	0.47 ± 0.07 ^c^	0.05 ± 0.02 ^c^	0.18 ± 0.01 ^c^
**Sucrose (%)**	0.51 ± 0.51 ^a^	1.00 ± 0.13 ^a^	0.15 ± 0.19 ^a^	0.15 ± 0.23 ^a^	0.17 ± 0.29 ^a^	0.66 ± 0.31 ^a^	0.29 ± 0.21 ^a^
**Rafinose (%)**	0.015 ± 0.01 ^c^	nd	0.27 ± 0.10 ^a^	nd	0.41 ± 0.08 ^c^	0.06 ± 0.09 ^c^	0.07 ± 0.07 ^c^
**Melezitose (%)**	0.13 ± 0.03 ^c^	0.09 ± 0.04 ^c^	nd	nd	0.20 ± 0.06 ^c^	0.41 ± 0.03 ^c^	nd
**Panose (%)**	0.05 ± 0.01 ^c^	0.02 ± 0.01 ^c^	nd	nd	nd	nd	nd
**Granulation indexes**							
**Total sugar content**	73.73 ± 0.21 ^a^	83.67 ± 2.25 ^d^	74.64 ± 3.95 ^d^	68.02 ± 4.76 ^e^	70.6 ± 2.98 ^d^	73.82 ± 5.43 ^e^	73.34 ± 3.19 ^d^
**F + G**	65.01 ± 1.04 ^d^	75.83 ± 0.71 ^b^	67.68 ± 0.82 ^d^	61.46 ± 0.53 ^b^	62.47 ± 0.9 ^b^	68.18 ± 1.25 ^d^	67.2 ± 0.92 ^d^
**F/G ratio**	1.31 ± 0.07 ^c^	1.12 ± 0.06 ^c^	1.32 ± 0.06 ^c^	1.27 ± 0.05 ^c^	1.35 ± 0.02 ^c^	1.30 ± 0.01 ^c^	1.00 ± 0.03 ^c^

Mean ± standard deviation values in the same column with different superscript letters (a–e) are significantly different (*p* < 0.05); (F + G) summation of fructose and glucose; (F/G) ratio of fructose to glucose and nd: not identified.

**Table 2:** Mean values of mineral elements and heavy metals (mg/kg) in the honey types.

	Elements (mg/kg)								
Honey Type	Potassium	Calcium	Magnesium	Iron	Zinc	Manganese	Copper	Cadmium	Lead
**Jujube**	395.53 ± 20.87 ^f^	107.81 ± 8.37 ^d^	38.66 ± 9.73 ^d^	16.65 ± 1.52 ^b^	5.50 ± 0.58 ^a^	2.46 ± 1.13 ^b^	1.22 ± 0.87 ^a^	0.0019 ± 0.01 ^a^	0.17 ± 0.027 ^a^
**Multifloral**	452.66 ± 18.34 ^f^	145.24 ± 10.78 ^d^	33.37 ± 5.23 ^c^	23.2 ± 0.86 ^a^	2.40 ± 0.18 ^a^	3.08 ± 1.08 ^b^	1.23 ± 0.75 ^a^	0.018 ± 0.09 ^a^	0.19 ± 0.012 ^a^
**Citrus**	305.90 ± 14.55 ^e^	69.56 ± 7.15 ^c^	16.57 ± 5.98 ^c^	12.57 ± 0.74 ^a^	2.19 ± 0.13 ^a^	2.12 ± 0.87 ^a^	0.99 ± 0.45 ^a^	0.0017 ± 0.04 ^a^	0.18 ± 0.042 ^a^
**Eucalyptus**	115.37 ± 17.22 ^f^	150 ± 5.44 ^c^	33.75 ± 10.27 ^d^	5.45 ± 1.67 ^b^	5.08 ± 0.21 ^a^	3.27 ± 1.54 ^b^	1.30 ± 0.60 ^a^	0.011 ± 0.03 ^a^	0.13 ± 0.040 ^a^
**Thyme**	330.52 ± 18.16 ^f^	40.33 ± 4.12 ^b^	27.23 ± 4.12 ^b^	4.67 ± 0.63 ^a^	2.88 ± 0.30 ^a^	2.98 ± 0.97 ^a^	1.27 ± 0.37 ^a^	0.0014 ± 0.07 ^a^	0.09 ± 0.002 ^a^
**Carob**	180.20 ± 14.44 ^e^	29.68 ± 6.18 ^c^	24.78 ± 3.34 ^b^	7.30 ± 0.85 ^a^	1.49 ± 0.19 ^a^	2.38 ± 0.73 ^a^	1.31 ± 0.29 ^a^	0.0016 ± 0.01 ^a^	0.10 ± 0.002 ^a^
**Rosemary**	49.31 ± 14.56 ^e^	12.33 ± 8.31 ^d^	12.03 ± 6.55 ^c^	2.15 ± 0.39 ^a^	12.46 ± 0.12 ^a^	1.45 ± 0.64 ^a^	3.32 ± 0.92 ^a^	0.017 ± 0.04 ^a^	0.14 ± 0.011 ^a^

Mean ± standard deviation values in the same column with different superscript letters (a–f) are significantly different (*p* < 0.05).

**Table 3:** Principal component analysis of elemental composition: eigenvalues, variance explained, and factor loadings.

Variance Explained Factor Loading												
PC	Eigenvalues	% of Variance	% Cumulative	K	Ca	Mg	Fe	Zn	Mn	Cu	Pd	Cd
**1**	5.031	56.143	56.143	0.751	0.794	0.768	0.791	–0.598	0.655	–0.738	0.022	0.631
**2**	1.751	19.586	75.730	–0.311	0.407	0.158	0.134	0.645	0.138	0.509	0.579	0.540

## Data Availability

The original contributions presented in this study are included in the article/Supplementary Materials. Further inquiries can be directed to the corresponding authors.

## Supplementary Materials

The following supporting information can be downloaded at: https://www.mdpi.com/article/10.3390/molecules30010150/s1, Table S1 lists the types of honeys tested and provides regional data, further contextualizing the geographical origins of each sample. Table S2 presents the sugar profile analysis of various honey types using gas chromatography-flame ionization detection (GC-FID), offering a comprehensive view of the sugar content across the different samples Additionally, the chromatograms shown in Figures S1–S7. illustrate the distinct sugar profiles of individual honey types, including jujube, multifloral, citrus, eucalyptus, thyme, carob, and rosemary honeys. These figures visually represent the unique chromatographic patterns for each honey type, showcasing variations in their sugar composition and supporting the finding of the main analysis.
