# Supplementary material for: Unveiling the Mineral and Sugar Richness of Moroccan Honeys: A Study of Botanical Origins and Quality Indicators
Source: Molecules. 2025 Jan 2;30(1):150. doi: 10.3390/molecules30010150 (PMC11721970; doi:10.3390/molecules30010150)
Supplement: Supplementary file 1 [file molecules-30-00150-s001.zip › molecules-3336930-supplementary.pdf]

Table S1. Sugar Profile Analysis of Various Honey Types by GC-FID

Honey types			Jujube	Multifloral	Citrus	Eucalyptus	Thyme	Carob	Rosemary
N°	Element	t_R (min)	Area (µV.s)						
1	Fructose	8.1	72226	76524	73893	70451	71217	74103	69819
2	Glucose	8.5	59520	65646	61978	59122	58885	65598	69678
3	Sucrose	18.3	12560	16091	8540	8544	9058	11876	3
4	Turanose	26.4	20297	18502	16050	14010	18050	14879	15950
5	Maltose	27.1	28298	39138	48298	26400	45880	26357	27391
6	Isomaltose	32.9	13259	11378	11089	10991	12171	2312	9176
7	Raffinose	42.5	3398	n.d	11686	n.d	12005	2465	2652
8	Erlose	43	19108	13056	10832	19889	12658	11259	12912
9	Melezitose	43.4	78791	2950	n.d	n.d	10092	11992	n.d
10	Panose	48 .2	2309	1798	n.d	n.d	n.d	n.d	n.d

Table S2. Types of honeys tested along with geographical origins

**Codes**	**Honey types**	**Geographical Origin**
S1	Jujube	Oujda (Angad)
S2	Jujube	Berkane (Tafoughalt)
S3	Jujube	Jerada (Guenfouda)
S4	Jujube	Nador (Al Aaroui)
S5	Jujube	Taourirt (Naïma)
S6	Multifloral	Oujda (Angad)
S7	Multifloral	Berkane (Tafoughalt)
S8	Multifloral	Jerada (Guenfouda)
S9	Multifloral	Nador (Al Aaroui)
S10	Multifloral	Taourirt (Naïma)
S11	Citrus	Oujda (Angad)
S12	Citrus	Berkane (Tafoughalt)
S13	Citrus	Jerada (Guenfouda)
S14	Citrus	Nador (Al Aaroui)
S15	Citrus	Taourirt (Naïma)
S16	Eucalyptus	Oujda (Angad)
S17	Eucalyptus	Berkane (Tafoughalt)
S18	Eucalyptus	Jerada (Guenfouda)
S19	Eucalyptus	Nador (Al Aaroui)
S20	Eucalyptus	Taourirt (Naïma)
S21	Thyme	Oujda (Angad)
S22	Thyme	Berkane (Tafoughalt)
S23	Thyme	Jerada (Guenfouda)
S24	Thyme	Nador (Al Aaroui)
S25	Thyme	Taourirt (Naïma)
S26	Carob	Oujda (Angad)
S27	Carob	Berkane (Tafoughalt)
S28	Carob	Jerada (Guenfouda)
S29	Carob	Nador (Al Aaroui)
S30	Carob	Taourirt (Naïma)
S31	Rosemary	Oujda (Angad)
S32	Rosemary	Berkane (Tafoughalt)
S33	Rosemary	Jerada (Guenfouda)
S34	Rosemary	Nador (Al Aaroui)
S35	Rosemary	Taourirt (Naïma)

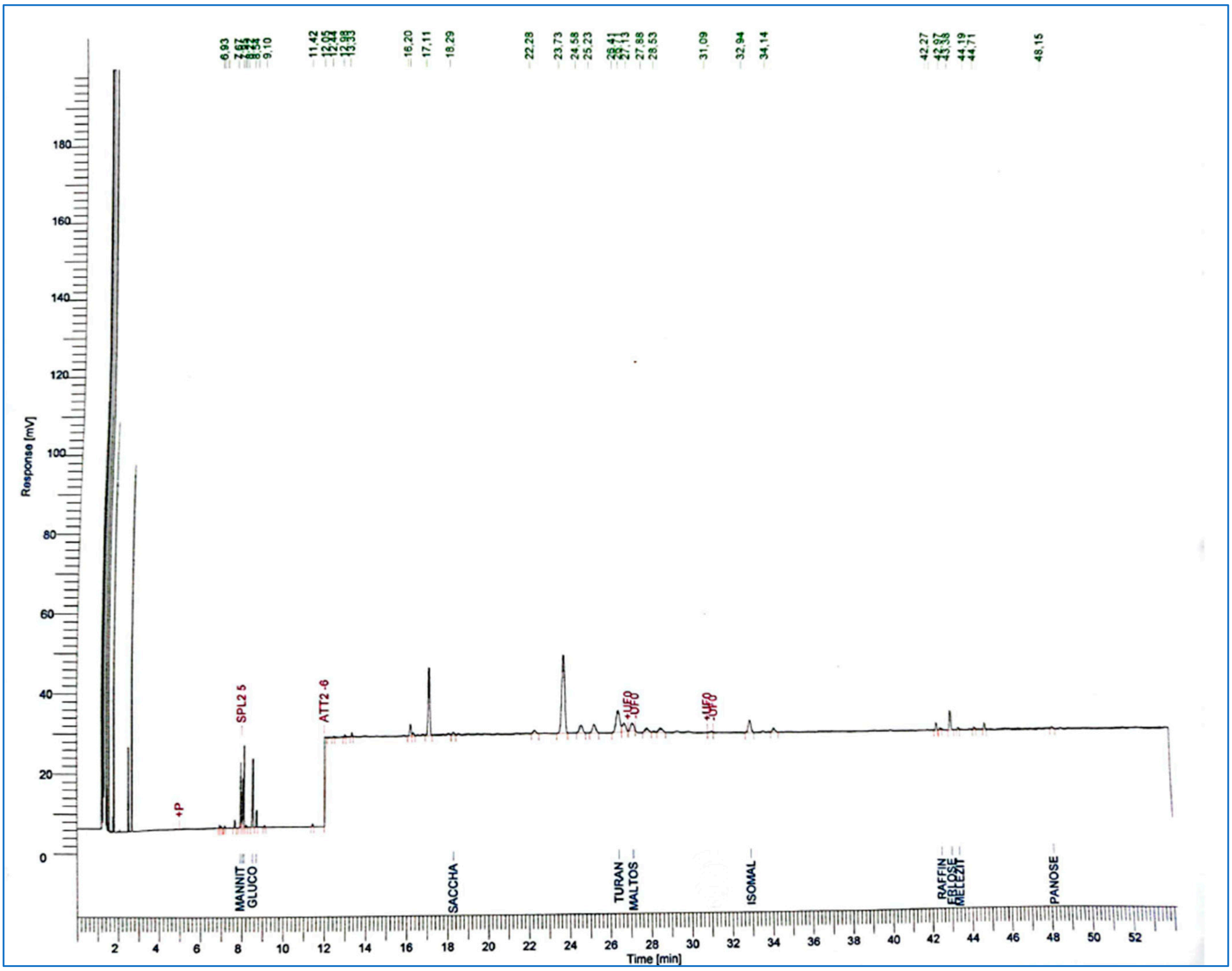

Figure S1. GC Chromatogram of *Jujube* honey

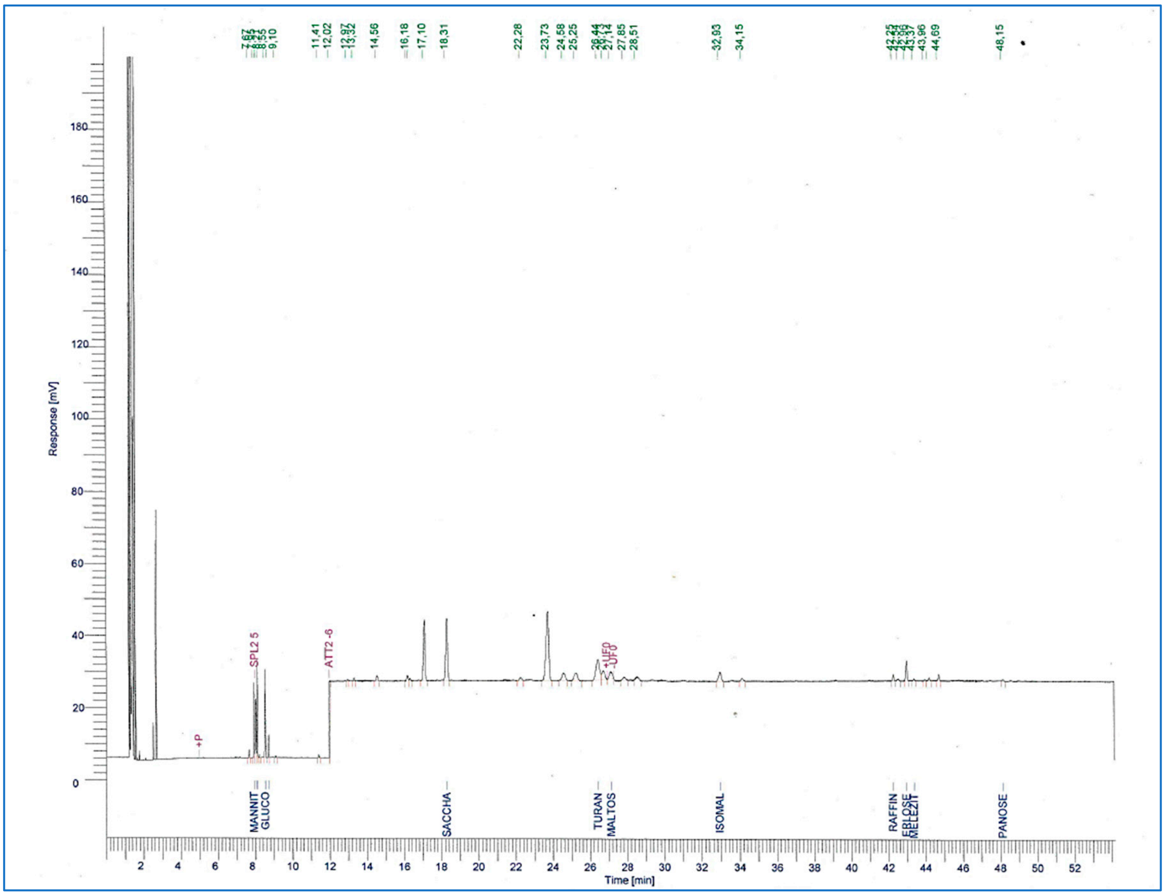

Figure S2. GC Chromatogram of *Multifloral* honey

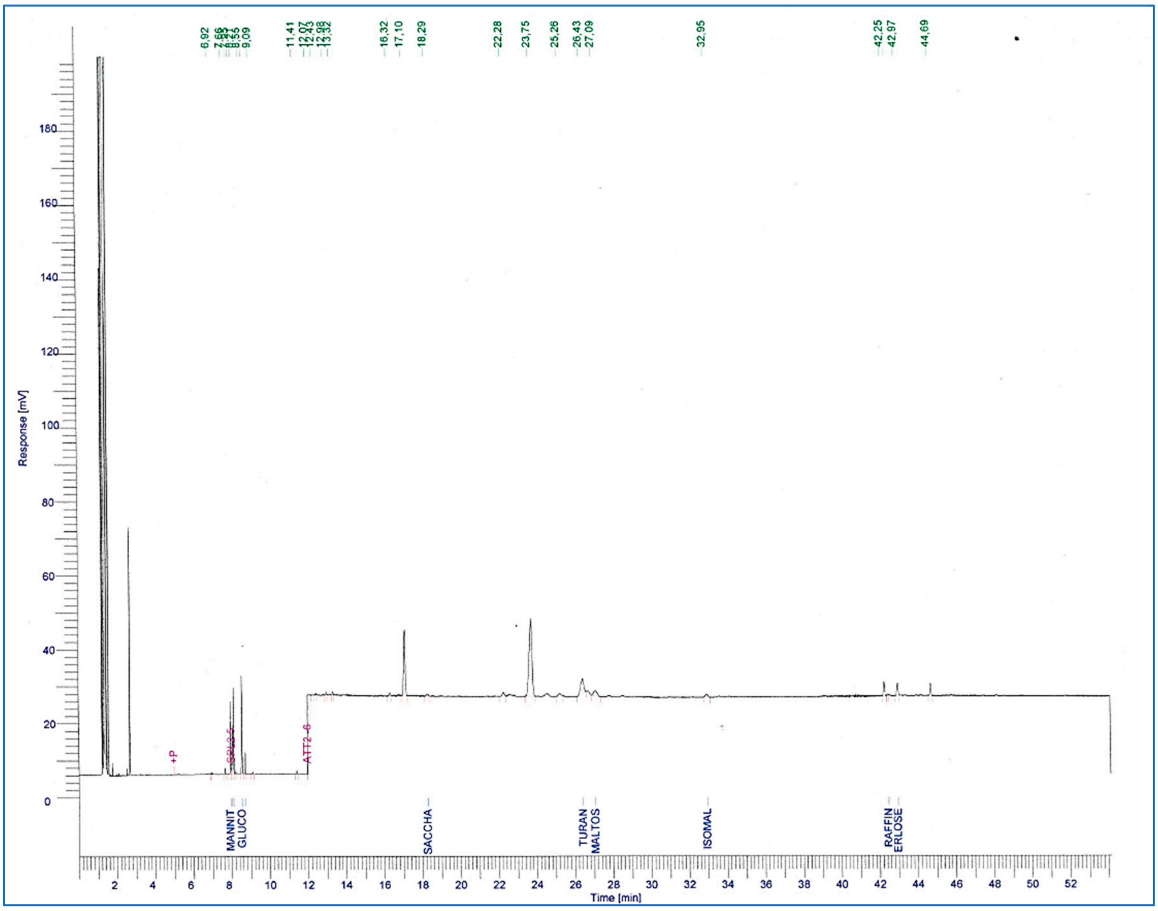

Figure S3. GC Chromatogram of *Citrus* honey

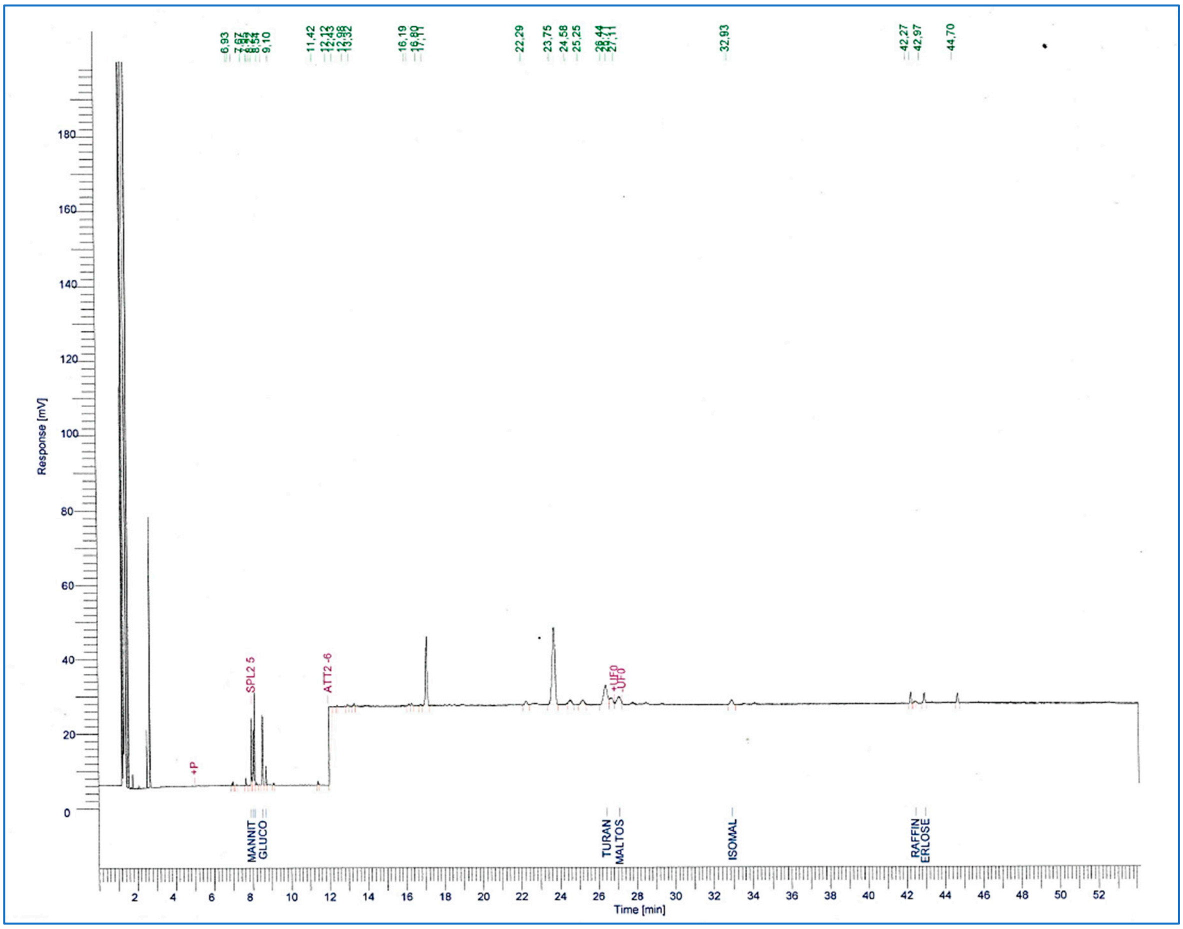

Figure S4. GC Chromatogram of *Eucalyptus* honey

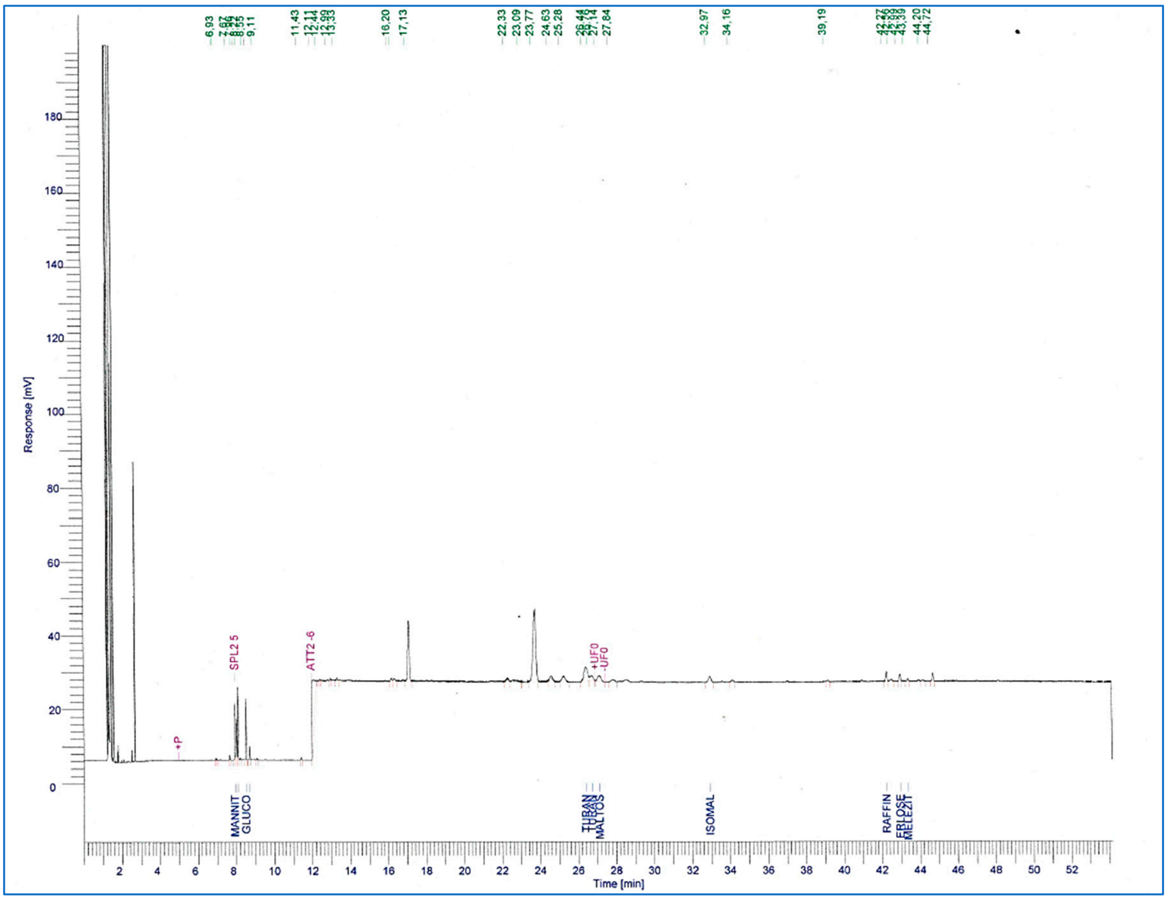

Figure S5. GC Chromatogram of *Thyme* honey

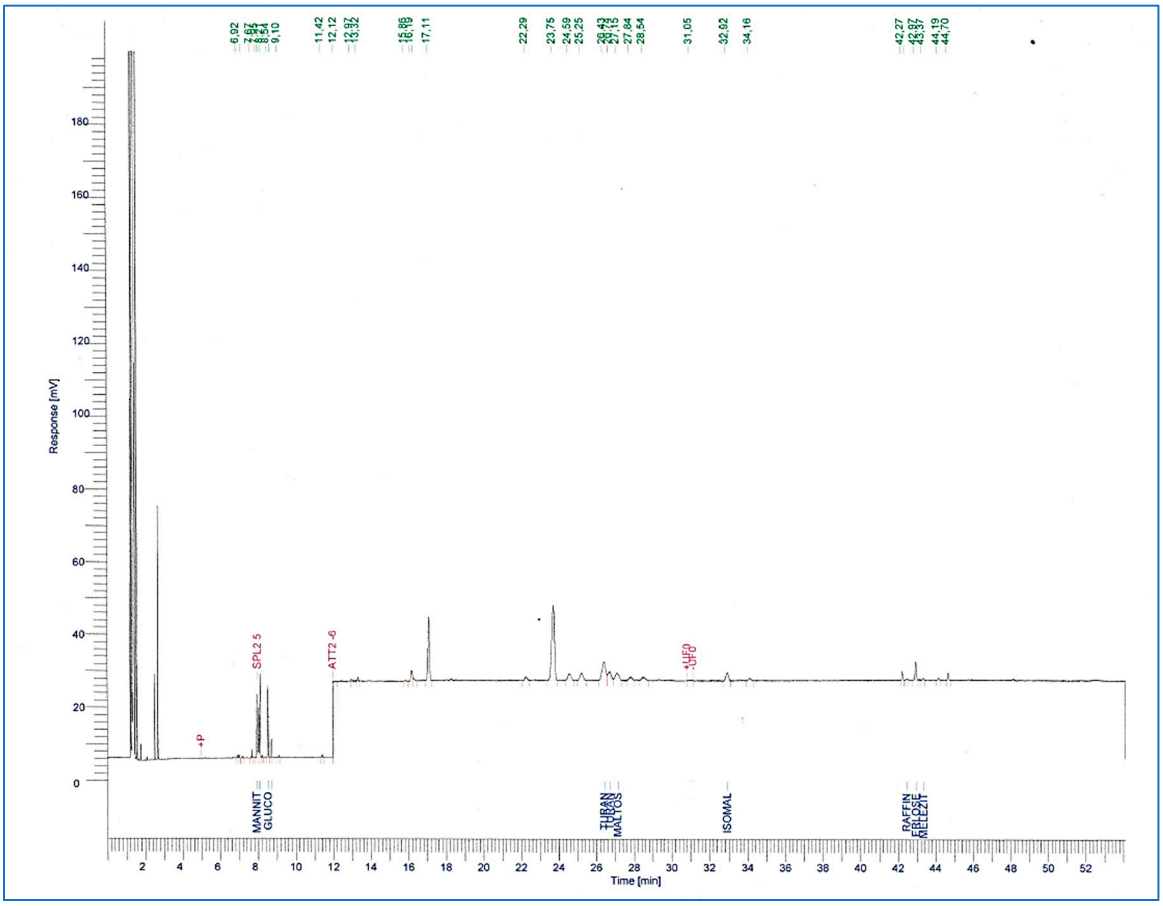

Figure S6. GC Chromatogram of *Carob* honey

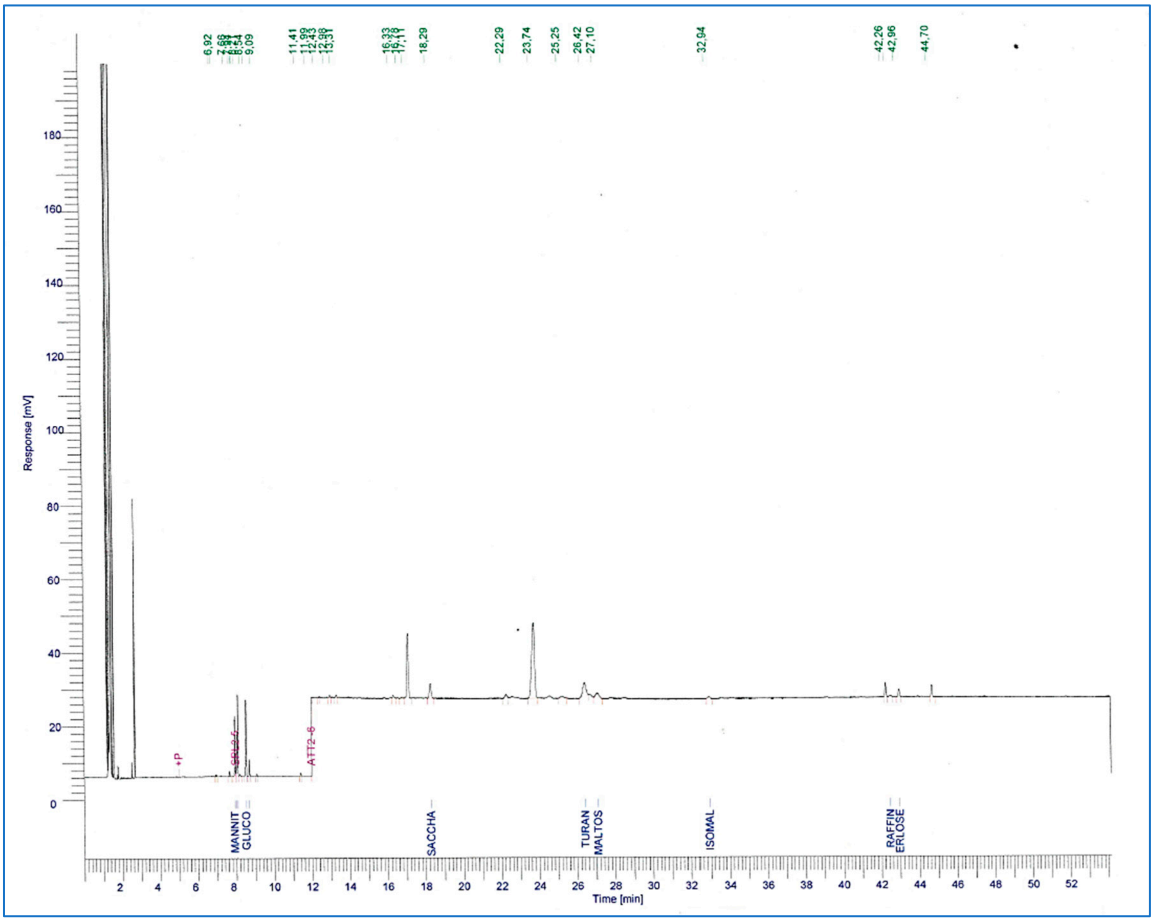

Figure S7. GC Chromatogram of *Rosemary* honey
